# Clinical features of gout in adult patients with type Ia glycogen storage disease: a single-centre retrospective study and a review of literature

**DOI:** 10.1186/s13075-021-02706-5

**Published:** 2022-02-26

**Authors:** Na Xu, Xinxin Han, Yun Zhang, Xiaoming Huang, Weiguo Zhu, Min Shen, Wen Zhang, Chen Jialin, Min Wei, Zhengqing Qiu, Xuejun Zeng

**Affiliations:** 1 Department of family medicine & Division of General Internal Medicine, Department of medicine, Peking Union Medical College Hospital, Chinese Academy of Medical Science & Peking Union Medical College, State Key Laboratory of Complex Severe and Rare Diseases (Peking Union Medical College Hospital), Beijing, China; 2 Department of Rheumatology, Peking Union Medical College Hospital, Chinese Academy of Medical Science & Peking Union Medical College, Beijing, China; 3 Department of Pediatrics, Peking Union Medical College Hospital, Chinese Academy of Medical Sciences & Peking Union Medical College, Beijing, China

**Keywords:** Glycogen storage disease, Type Ia, Gout, Hyperuricaemia

## Abstract

**Background:**

This study aimed to explore the clinical features of gout in adult patients with glycogen storage disease type Ia (GSD Ia).

**Methods:**

Ninety-five adult patients with GSD Ia admitted to Peking Union Medical College Hospital were retrospectively analysed. A clinical diagnosis of GSD Ia was confirmed in all patients through gene sequencing. All patients had hyperuricaemia; 31 patients complicated with gout were enrolled, and 64 adult GSD Ia patients with asymptomatic hyperuricaemia were selected as a control group during the same period. Clinical characteristics were analysed and compared between the two groups.

**Results:**

Thirty-one of the 95 patients had complications of gout (median age, 25 years; 11 (35.5%) females). All 31 patients had hepatomegaly, abnormal liver function, fasting hypoglycaemia, hyperuricaemia, hyperlipaemia, and hyperlacticaemia. A protuberant abdomen, growth retardation, recurrent epistaxis, and diarrhoea were the most common clinical manifestations. Among these 31 patients, 10 patients (32.3%) had gout as the presenting manifestation and were diagnosed with GSD Ia at a median time of 5 years (range, 1–14) after the first gout flare. The median age of gout onset was 18 years (range, 10–29). Fifteen of the 31 GSD Ia-related gout patients were complicated with gouty tophi, which has an average incidence time of 2 years after the first gouty flare. The mean value of the maximum serum uric acid (SUA) was 800.5 μmol/L (range, 468–1068). The incidence of gout in adult GSD Ia patients was significantly associated with the initial age of regular treatment with raw corn starch, the proportion of urate-lowering therapy initiated during the asymptomatic hyperuricaemic stage, maximum SUA level, and mean cholesterol level.

**Conclusions:**

Determination of GSD Ia should be performed for young-onset gout patients with an early occurrence of gouty tophi, especially in patients with hepatomegaly, recurrent hypoglycaemia, or growth retardation. Early detection and long-term regulatory management of hyperuricaemia, in addition to early raw corn starch and lifestyle intervention, should be emphasized for GSD Ia patients in order to maintain good metabolic control.

**Trial registration:**

Retrospectively registered.

## Background

Gout, a recognized complication of hyperuricaemia (HUA), is the most common type of inflammatory arthritis in adults, caused by the deposition of monosodium urate (MSU) crystals in joints or adjacent soft tissues. Clinical manifestations include painful attacks of acute gouty arthritis, the formation of tophaceous MSU crystal deposits, chronic joint damage, renal stone formation, and potential renal insufficiency, which affect the quality and longevity of life [1–3]. Moreover, increasing evidence indicates that gout is associated with various chronic diseases including diabetes mellitus, hypertension, obesity, and increased risk of cardiovascular diseases [4–6].

Gout is more common in men and increases with age; it is uncommon in men younger than 40 years and premenopausal women [7–9]. For patients presenting with early-onset gout, screening is needed for potential causes. Glycogen storage disease (GSD), a unique category of inherited metabolic disorders, is one of the rarer causes of gout in young patients [10, 11]. Glycogen storage disease type Ia (GSD Ia) is an autosomal recessive disorder caused by mutations in the glucose-6-phosphatase catalytic subunit (*G6PC*) gene leading to a deficiency of the glucose-6-phosphatase catalytic unit (G6Pase-α) enzyme [12]. Mutations in this gene result in the inhibition of glucose production and accumulation of glycogen and fat in the liver, kidney, and intestine. The overall incidence of GSD Ia is considered to be 1 in 100,000. Typical manifestations include hepatomegaly, hypoglycaemia, growth retardation, lactic acidosis, hypertriglyceridaemia, and HUA, which usually manifest in the infantile period [13–15]. In addition, hepatocellular adenoma and renal damage are frequent complications that develop later in life [16–18]. A delayed diagnosis and inappropriate interventions can lead to many complications, such as growth failure, refractory gout, renal failure, and hepatocellular carcinoma.

HUA is almost always present in patients with GSD Ia from childhood. Two underlying mechanisms act in combination to elevate the serum uric acid (SUA) levels in GSD Ia patients: decreased renal excretion of urate secondary to lactic acadaemia and ketonaemia and elevated production of uric acid due to the routing of glucose-6-phosphatase to the pentose pathway [19, 20]. As the life expectancy of individuals with GSD Ia has improved considerably, most children reach the age of adulthood. Gout is not uncommon in adult patients as it is one of the late complications that impact the quality of life, especially in those with insufficient metabolic control. However, these patients may be initially misdiagnosed as having primary gout, leading to the underlying secondary cause of GSD being overlooked; thus, diagnostic delays may occur. Understanding the clinical features of gout in GSD Ia is important for early diagnosis and treatment. In addition, identifying the risk factors of gouty attacks in GSD Ia patients may help physicians establish interventions at earlier stages of the disease, to prevent the disease from progressing. To date, well-described studies of the clinical features and risk factors of gout in adult GSD Ia patients are lacking, as less than 20 such cases have been reported in the English literature thus far [15]. Therefore, we conducted this study to investigate the clinical features and risk factors of gout in adult patients with GSD Ia.

## Methods

### Study design and participants

**Fig. 1:**
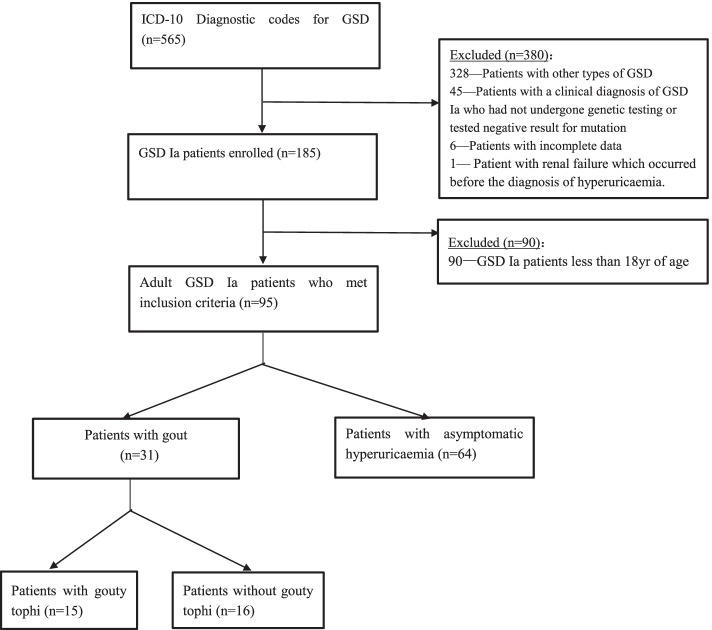
Flow chart of the patients included in the study. GSD, glycogen storage disease; GSD Ia, glycogen storage disease type Ia

The approach used for the development of the study cohort is summarized in Fig. 1. We retrospectively reviewed the medical records of 95 adult GSD Ia patients who had been admitted to Peking Union Medical College Hospital (PUMCH) between 1 January 1999 and 30 December 2018. All admitted patients were aged 18 years old and above. All patients had HUA. Of these patients, a total of 31 adult GSD Ia patients who had complications of gout were enrolled and analysed retrospectively, whereas 64 adult GSD Ia patients with asymptomatic HUA were selected as controls during the same period. Clinical diagnosis of GSD Ia was confirmed in all patients through targeted analysis of *G6PC* gene sequencing. HUA was defined as a SUA level higher than 7.0 mg/dL (420 μmol/L) for males and 6.0 mg/dL (357 μmol/L) for females. Gout was diagnosed based on the 2015 European League Against Rheumatism (EULAR)/American College of Rheumatology criteria. Gouty attack was defined as documentation of the acute onset of joint pain with some clinical features of inflammation, or treatment in keeping with that used for an acute flare, including non-steroidal anti-inflammatory drugs, colchicine, or prednisone [21]. Renal insufficiency was defined as an estimated glomerular filtration rate (eGFR) < 60 mL/min/1.73 m^2^. The exclusion criteria included patients with incomplete data or HUA secondary to other causes, including drugs, malignancies, or renal failure which occurred before the onset of HUA.

Clinical characteristics were analysed and compared between the two groups: the gout group (GSD Ia patients with gout) and the control group (GSD Ia patients with asymptomatic HUA). In addition, patients in the gout group were assigned to one of two subgroups: the tophus group (patients with gouty tophi) and the non-tophus group (patients without gouty tophi). Clinical characteristics were analysed and compared between the two subgroups. Moreover, a total of 93 cases were randomly selected from 729 primary gout patients aged between 18 and 45 years in PUMCH during the same period, and the clinical features of gout were compared between patients with GSD Ia-related gout and primary gout. This study was reviewed and exempted by the Institutional Review Board of the Peking Union Medical College Hospital, Beijing, China.

### Statistical analysis

The software package, SPSS 21.0 (IBM, Armonk, NY, USA), was used to perform statistical analyses. Continuous variables with normal distribution are presented as mean ± standard deviation (SD). Non-normal variables are reported as median (range). Categorical variables are reported as absolute counts (percentage). The chi-square test and Fisher’s exact test were used to compare the categorical data, and the independent sample *t* test was used to compare the quantitative data between the groups. Logistic regression analysis was conducted to identify the risk factors of gout in adult GSD Ia patients. The strength of the association was estimated by odds ratios (ORs) with 95% confidence intervals (95% CIs). Hypothesis tests were two-tailed, with *P* < 0.05 considered statistically significant.

## Results

### Demographics of adult patients with GSD Ia-related gout

**Fig. 2:**
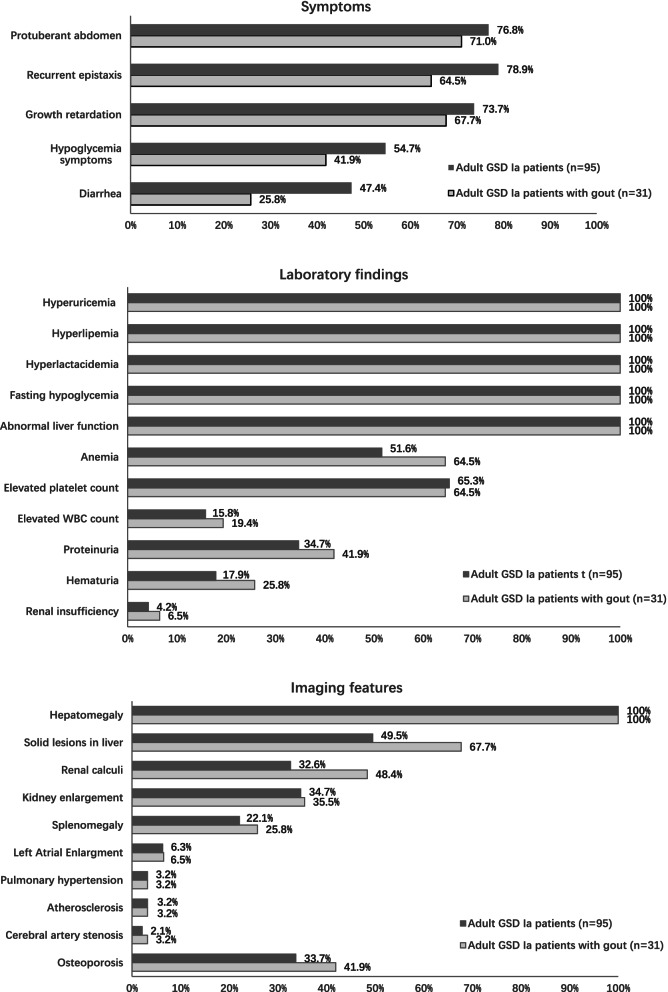
Clinical features of adult patients with GSD Ia. GSD Ia, glycogen storage disease type Ia

**Fig. 3:**
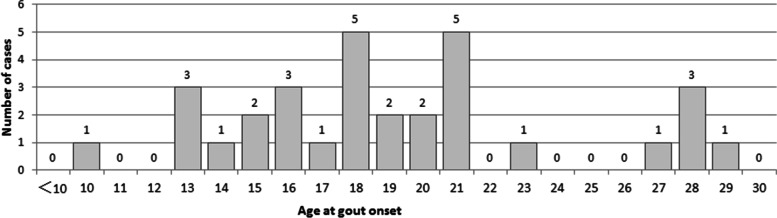
Age distribution of gout onset in patients with glycogen storage disease type Ia

The clinical features of the 95 adult GSD Ia patients in our cohort are shown in Fig. 2. Thirty-one (32.6%) of the 95 adult GSD Ia patients had complications of gout during the study period. Our cohort included 20 males and 11 females with a median age of 25 years (range, 19–41). The median age at the first episode of gouty arthritis was 18 years, ranging from 10 to 29 years old (Fig. 3). Of these 31 patients, 10 patients (32.3%) had gout as the presenting manifestation and were diagnosed with GSD Ia at a median time of 5 years (range, 1–14) after the first gouty attack. The median ages at symptom onset and diagnosis of GSD Ia were 2 (range, 0.5–15) and 12 years (range, 2–39), respectively. The mean time from the symptom onset to diagnosis of GSD Ia was 8 years (range, 1–37).

### Clinical characteristics of adult patients with GSD Ia-related gout

Among the 31 patients, six (19.4%) had a family history of GSD Ia and two (6.5%) had a family history of HUA. The initial symptoms of GSD Ia included a protuberant abdomen in 26 cases (64.5%), abnormal liver function in 2 cases (6.5%), recurrent epistaxis in 2 cases (6.5%), and hypoglycaemic symptoms in 1 case (3.1%). The common clinical manifestations included a protuberant abdomen in 22 cases (71.0%), growth retardation in 21 cases (67.7%), recurrent epistaxis in 20 cases (64.5%), and diarrhoea in 8 cases (25.8%). All 31 patients had hepatomegaly, abnormal liver function, fasting hypoglycaemia, HUA, and hyperlipaemia, as well as hyperlacticaemia.

The mean value of the maximum SUA before treatment was 800.5 μmol/L, ranging from 468 to 1068 μmol/L. Among the 31 patients, anaemia was found in 20 (64.5%) patients, in whom the mean level of the lowest haemoglobin was 84.3 mg/dL (range, 37–109). An elevated platelet count was found in 20 (64.5%) patients. In terms of renal involvement, haematuria was detected in eight (25.8%) patients, whereas proteinuria was detected in 13 (41.9%) patients, in whom the mean level of 24-h proteinuria was 1.3 g (range, 0.3–3.4). Two patients were complicated with chronic renal insufficiency, and the eGFR rate was 11 and 15 mL/min/1.73 m^2^, respectively. Renal calculi were found in 15 patients, with a median onset age of 23.3 years (range, 16–39). Kidney enlargement was noted in 11 cases (35.5%).

### Characteristics of gouty attacks and gouty tophi in adult patients with GSD Ia-related gout

The clinical features of the gouty attacks in the 31 adult GSD Ia patients are summarized in Table 1.

**Table 1:** Clinical features of gouty attack in GSD Ia patients

Variable	*n* = 31
Male	20 (64.5)
Gout symptom onset age, years	18 (10.29)
Gout symptom duration, years	5.0 ± 4.4
Site of first gouty attack	
Ankle	14 (45.2)
MTP1 joint	12 (38.7)
Knee	2 (6.5)
Elbows	1 (3.2)
Second MCP joint	1 (3.2)
PIP joint of the fifth finger	1 (3.2)
Involved joints at gouty attack	
Ankle joint	20 (64.5)
First MTP joint	14 (45.2)
Knee	13 (41.9)
MCP joint	8 (25.8)
Wrist	7 (22.6)
PIP joint	6 (19.4)
Elbows	3 (9.7)
PIP joint of the toe	2 (6.5)
Shoulder joint	1 (3.2)
Upper extremity involved	15 (48.4)
Number of involved joints	
1	7 (22.6)
2	4 (12.9)
3	5 (16.1)
4	4 (12.9)
≥ 5	11 (35.5)
Presence of tophi	15 (48.4)
Presence of renal calculi	15 (48.4)

Values are expressed as numbers (%), median (range), or mean ± standard deviation

*Abbreviation*: *GSD Ia* glycogen storage disease type Ia, *MTP1* first metatarsophalangeal, *MCP* metacarpophalangeal, *PIP* proximal interphalangeal, *eGFR* estimated glomerular filtration rate, *BMI* body mass index

The mean disease duration of gout was 5.0 years, and the average number of involved joints was 3.5 (range, 1–8). Among these 31 cases, 15 cases (48.4%) involved the upper limb joints. The sites commonly affected at the first gout flare included the ankles (45.2%) and first metatarsophalangeal (MTP1) joint (38.7%) and less commonly the knees (6.5%), elbows (3.2%), metacarpophalangeal (MCP) joint (3.2%), and proximal interphalangeal (PIP) joint (3.2%).

Fifteen out of 31 cases had gouty tophi. The incidence of gouty tophi within 1, 2, and 3 years after the first gouty flare accounted for 6.5%, 29%, and 32.2% of cases, respectively. The median onset age of gouty tophi was 21.5 years (range, 14–33). The mean time from the first episode of gouty arthritis to the development of gouty tophi was 2 years (range, 1–6). The sites of gouty tophi were distributed in the auricle, MTP1, wrist, MCP, PIP, elbow joints, hands, feet, hips, back, and limbs. Among the 15 patients with gouty tophi, 10 had multiple tophi distributed in three or more sites, and the maximum diameter of the tophi was 4–5 cm. Two patients underwent surgical removal of the tophi due to its large size and influence on joint function.

We further analysed and compared the clinical features between the two subgroups of adult GSD Ia-related gout patients with and without gouty tophi; the results are shown in Table 2.

**Table 2:** Clinical features of the two subgroups of adult GSD Ia-related gout patients with and without gouty tophi

Variable	Patients with gouty tophi (***n*** = 15)	Patients without gouty tophi (***n*** = 16)	***P***
Male	11 (73.3)	9 (56.3)	0.320
Family history of HUA	1 (6.7)	1 (6.3)	0.962
Age, years	28.1 ± 6.6	23.4 ± 5.4	0.036*
Age at GSD diagnosis, years	13 (2,39)	11 (3,30)	0.147
Gout symptom onset age, years	18 (10,29)	19 (13,28)	0.383
Gout symptom duration, years	6.1 ± 5.4	3.8 ± 2.1	0.042*
Number of involved joints	4.6 ± 1.5	2.4 ± 1.8	0.001*
Upper extremity involved	12 (80.0)	3 (18.8)	0.014*
Renal calculi	11 (73.3)	4 (25.0)	0.005*
Haematuria	5 (33.3)	3 (18.8)	0.354
Proteinuria	5 (33.3)	8 (50.0)	0.347
Renal insufficiency	1 (6.7)	1 (6.3)	0.962
eGFR	119.1 ± 53.4	141.8 ± 46.7	0.218
Kidney enlargement	5 (33.3)	6 (37.5)	0.901
Anaemia	10 (66.7)	10 (62.5)	0.809
Hepatic adenoma	12 (80.0)	8 (50.0)	0.081
SUA, umol/L	812.1 ± 126.9	789.7 ± 137.9	0.641
TG, mmol/L	10.8 ± 6.9	13.8 ± 13.0	0.430
TC, mmol/L	6.3 ± 2.3	7.8 ± 3.2	0.146
LDL-C, mmol/L	3.0 ± 0.7	3.5 ± 1.5	0.268
LA, mmol/L	9.4 ± 4.3	8.9 ± 3.1	0.722
FBS, mmol/L	2.9 ± 0.7	2.9 ± 0.7	0.909
Height, cm	159.2 ± 6.1	159.5 ± 10.2	0.932
BMI, kg/m^2^	21.2 ± 4.0	21.5 ± 4.3	0.894

Values are expressed as numbers (%), median (range) or mean ± standard deviation

*Abbreviation*: *HUA* hyperuricaemia, *GSD Ia* glycogen storage disease type Ia, *eGFR* estimated glomerular filtration rate, *SUA* serum uric acid, *TG* triglycerides, *TC* total cholesterol, *LDL-C* low-density lipoprotein cholesterol, *LA* lactic acid, *FBS* fasting blood sugar, *BMI* body mass index

*P < 0.05

The mean age, mean disease duration of gout, average number of involved joints, and proportion of upper limb joint involvement in the tophus group were significantly higher than those in the non-tophus group (*P* < 0.05). There were no significant differences between the two groups in terms of sex, family history, the median age of gout onset, and the proportion of haematuria, proteinuria, renal insufficiency, renal enlargement, anaemia, liver lesions, SUA level, triglycerides (TG), total cholesterol (TC), low-density lipoprotein cholesterol (LDL-C), lactic acid and fasting blood glucose, body mass index, and eGFR rate.

### Treatments and outcomes of adult patients with GSD Ia-related gout

All 31 patients were treated with raw corn starch after being diagnosed with GSD Ia. The symptoms of acute gouty arthritis could be relieved after anti-inflammatory treatment in all patients; however, the episodes of gouty flares gradually became more frequent before urate-lowering therapy (ULT), even on a low-purine diet. Before hospital admission, 7 out of 31 patients had received allopurinol since the stage of asymptomatic HUA. However, SUA levels reduced to 490 μmol/L in one patient (allopurinol 200 mg/day) and 520 μmol/L in another patient (allopurinol 300 mg/day), whereas five patients self-discontinued allopurinol. Twenty-four out of 31 patients received ULT after gouty attack; the median time from gouty onset to ULT was 2 years (range, 0.5–17); however, six patients self-discontinued ULT.

After hospital admission, all patients were treated with a combination of oral raw corn starch, ULT, and a low-purine and low-fat diet on admission to our hospital, as well as through patient education and the provision of information on appropriate lifestyle, including advice regarding weight loss in patients with obesity and the avoidance of alcoholic and sugary drinks, heavy meals, and excessive consumption of meat and seafood. The dosage of corn starch was 1.75–2.5 g per kilogram of body weight, every 6 h, and blood glucose level was maintained at more than 70 mg/dL. ULT included allopurinol at daily doses of 200–400 mg in 29 patients and febuxostat at daily doses of 40–60 mg in 2 patients who were complicated with chronic renal insufficiency. Additionally, 12 patients who were complicated with osteoporosis were given calcium and vitamin D supplementation. Regarding the nine patients with hepatic adenoma, two patients recovered well after surgical treatment, five patients underwent transcatheterial embolisation chemotherapy, three patients were closely followed up, and the other three patients refused further treatment. One patient with hepatic carcinoma was transferred to a local hospital for treatment.

### Comparison of clinical data between adult GSD Ia-related gout patients and controls

Table 3 details the clinical and laboratory data of adult GSD Ia-related gout and control patients. The patients in the gout group had a significantly higher incidence of renal calculus (48.4% vs 25.0%), maximum SUA level (800.5 ± 131.0 μmol/L vs 648.6 ± 120.7 μmol/L), mean cholesterol level (7.3 ± 4.1 mmol/L vs 5.8 ± 1.7 mmol/L), and platelet level (444.5 ± 198.4×10^9^/L vs 376.1 ± 117.2×10^9^/L) than the control group, respectively. Compared with the control group, patients in the GSD-related gout group showed a higher mean age at onset and diagnosis for GSD. The initial age of regular treatment with raw corn starch (16.1 ± 9.1 vs 11.7 ± 9.1 years, *P* = 0.003) in the gout group was later than that in the control group, whereas the proportion of ULT initiated during the asymptomatic HUA stage was lower (22.6% vs 62.5%, *P* < 0.001).

**Table 3:** Clinical features of the two groups of adult GSD Ia patients with and without gout

Variable	Total (*n* = 95)	Gout group (*n* = 31)	Controls (*n* = 64)	*P*
Male	57 (60.0)	20 (64.5)	37 (57.8)	0.532
Age, years	22 (18, 41)	25 (19, 41)	21.5 (18, 30)	0.098
Age at GSD onset, years	2 (0.1, 15)	2 (0.5, 15)	1.3 (0.1, 9)	0.01*
Age at GSD diagnosis, years	11 (1, 39)	12 (2, 39)	11 (1, 23)	0.047*
Mean time from the onset to diagnosis, years of GSD Ia	9 (0.5, 37)	8 (1, 37)	9 (0.5, 23)	0.391
Family history of GSD	22 (23.2)	6 (19.4)	16 (25.0)	0.541
Family history of HUA	2 (2.1)	2 (6.5)	0 (0)	0.104
Clinical features				
Symptoms				
Protuberant abdomen	73 (76.8)	22 (71.0)	51 (79.7)	
Recurrent epistaxis	75 (78.9)	20 (64.5)	55 (85.9)	0.016*
Growth retardation	70 (73.7)	21 (67.7)	49 (76.6)	0.360
Hypoglycaemia symptoms	52 (54.7)	13 (41.9)	39 (60.9)	0.081
Diarrhoea	45 (47.4)	8 (25.8)	37 (57.8)	0.003*
Hepatic involvement				
Hepatomegaly	95 (100)	31 (100)	64 (100)	1.000
Abnormal liver function	95 (100)	31 (100)	64 (100)	1.000
Hepatic adenoma	46 (48.4)	20 (64.5)	26 (40.6)	0.029*
Hepatocellular carcinoma	1 (1.1)	1 (3.2)	0 (0)	0.326
Splenomegaly	21 (22.1)	8 (25.8)	13 (20.3)	0.545
Abnormal haematological finding				
Anaemia	49 (51.6)	20 (64.5)	29 (45.3)	0.079
Elevated platelet count	62 (65.3)	20 (64.5)	42 (65.6)	0.915
Elevated white blood cell count	15 (15.8)	6 (19.4)	9 (14.1)	0.507
Renal complication				
Renal calculi	31 (32.6)	15 (48.4)	16 (25.0)	0.023*
Kidney enlargement	33 (34.7)	11 (35.5)	22 (34.4)	0.915
Proteinuria	33 (34.7)	13 (41.9)	20 (31.3)	0.305
Haematuria	17 (17.9)	8 (25.8)	9 (14.1)	0.161
Renal insufficiency	4 (4.2)	2 (6.5)	2 (3.1)	0.449
Metabolic complications/comorbidities				
Hyperuricaemia	95 (100)	31 (100)	64 (100)	1.000
Hyperlipaemia	95 (100)	31 (100)	64 (100)	1.000
Hyperlacticaemia	95 (100)	31 (100)	64 (100)	1.000
Fasting hypoglycaemia	95 (100)	31 (100)	64 (100)	1.000
BMI > 24 (kg/m^2^)	8 (8.4)	5 (16.1)	3 (4.7)	0.060
Diabetes mellitus	2 (2.1)	1 (3.2)	1 (1.6)	0.596
Left atrial enlargement	6 (6.3)	2 (6.5)	4 (6.3)	0.970
Pulmonary hypertension	3 (3.2)	1 (3.2)	2 (3.1)	0.979
Atherosclerosis	3 (3.2)	1 (3.2)	2 (3.1)	0.979
Anterior/middle cerebral artery stenosis	2 (2.1)	1 (3.2)	1 (1.6)	0.596
Osteoporosis	32 (33.7)	13 (41.9)	19 (29.7)	0.236
Laboratory findings				
SUA, μmol/L				
Maximum value	700.4 ± 143.2	800.5 ± 131.0	648.6 ± 120.7	< 0.001*
Average value	542.8 ± 94.5	606.0 ± 109.6	525.0 ± 82.4	0.001*
LA, mmol/L				
Maximum value	10.2 ± 3.7	9.2 ± 3.7	10.7 ± 3.6	0.052
Average value	8.2 ± 2.8	7.2 ± 2.1	8.6 ± 2.9	0.055
TG, mmol/L				
Maximum value	12.6 ± 8.8	12.4 ± 10.5	12.7 ± 7.9	0.874
Average value	8.0 ± 4.1	7.4 ± 4.2	8.2 ± 4.1	0.456
TC, mmol/L				
Maximum value	7.0 ± 2.7	7.1 ± 2.9	7.0 ± 2.6	0.789
Average value	6.1 ± 2.5	7.3 ± 4.1	5.8 ± 1.7	0.033*
LDL-C, mmol/L	3.0 ± 1.1	3.3 ± 1.2	2.9 ± 1.1	0.119
Minimum FBS, mmol/L	2.8 ± 0.6	2.9 ± 0.7	2.7 ± 0.6	0.332
eGFR, mL/(min.1.73 m^2^)	134.6 ± 50.3	130.8 ± 50.6	136.5 ± 50.5	0.612
WBC, × 10^9^/L	7.9 ± 3.1	8.1 ± 3.1	7.7 ± 3.1	0.544
Hgb, g/L	103.4 ± 20.3	98.2 ± 24.3	106.0 ± 17.5	0.081
PLT, × 10^9^/L	399.4 ± 152.3	444.5 ± 198.4	376.1 ± 117.2	0.041*
ALT, μ/L	118.5 ± 75.4	105.8 ± 62.6	125.1 ± 80.9	0.250
AST μ/L	141.4 ± 99.7	117.4 ± 95.6	153.8 ± 100.2	0.099
GGT μ/L	155.6 ± 116.1	156.3 ± 140.4	155.3 ± 102.7	0.971
ALP μ/L	227.7 ± 135.2	226.9 ± 100.4	228.1 ± 50.5	0.969
Treatment				
Initial age of regular, years of treatment with raw corn starch	13.1 ± 6.9	16.1 ± 9.1	11.7 ± 9.1	0.003*
Proportion of ULT initiated during asymptomatic HUA stage	47 (49.5)	7 (22.6)	40 (62.5)	< 0.001*
Age of ULT initiated, years	16.3 ± 3.7	16.6 ± 1.8	16.2 ± 3.9	0.103
Proportion of treatment with lipid-lowering drugs [*n* (%)]	63 (66.3)	19 (61.3)	44 (68.8)	0.471

*Abbreviation*: *SUA* serum uric acid, *LA* lactic acid, *TG* triglycerides, *TC* total cholesterol, *LDL-C* low-density lipoprotein cholesterol, *FBS* fasting blood sugar, *eGFR* estimated glomerular filtration rate, *WBC* white blood cell, *Hgb* haemoglobin, *PLT* platelet, *ALT* alanine aminotransferase, *AST* aspartate aminotransferase, *GGT* gamma-GT, *ALP* alkaline phosphatase, *ULT* urate-lowering therapy

*P < 0.05

### Risk factors for gout in adult GSD Ia patients

In the multivariate analysis, the incidence of gout in adult GSD Ia patients was significantly associated with the initial age of regular treatment with raw corn starch (OR, 1.189; 95% CI, 0.009–1.400; *P* = 0.038), the proportion of ULT initiated during the asymptomatic HUA stage (OR, 0.057; 95% CI, 0.009–0.348; *P* = 0.002), maximum SUA level (OR, 1.012; 95% CI, 1.006–1.019, *P* < 0.01), and mean cholesterol level (OR, 1.288; 95% CI, 1.044–1.590; *P* = 0.018).

### Comparison of clinical data between adult patients with GSD Ia-related gout and primary gout

The GSD Ia-related gout group had a higher proportion of female patients (35.5% vs 2.2%, *P* < 0.001) and a younger age of gout onset (18 vs 28 years, *P* = 0.001) than the primary gout group. Compared with the patients with primary gout, patients with GSD Ia-related gout had a higher incidence of gouty tophi (48.4% vs 25.8%, *P* = 0.019), renal calculus (48.4% vs 21.5%, *P* = 0.004), proteinuria (41.9% vs 2.2%, *P* < 0.001), haematuria (25.8% vs 1.1%, *P* < 0.001), and a lower proportion of MTP1 as the initial involved joint (38.7% vs 59.1%, *P* = 0.048), as well as a lower BMI (21.4 ± 4.1 vs 27.4 ± 3.4 kg/m^2^, *P* = 0.001). In terms of laboratory examinations, the maximum SUA level (800.5 ± 131.0 vs 584.3 ± 112.8 μmol/L, *P* = 0.001), triglyceride level (12.4 ± 10.5 vs 2.9 ± 1.7 mmol/L, *P* = 0.003), and total cholesterol level (7.1 ± 2.9 vs 3.6 ± 1.5 mmol/L, *P* = 0.001) were significantly higher in patients with GSD Ia-related gout than in patients with primary gout. The comparisons of clinical data between adult patients with GSD Ia-related gout and primary gout is shown in Table 4.

**Table 4:** Clinical data of adult patients with GSD Ia-related gout and primary gout

Variable	GSD Ia-related gout (*n* = 31)	Primary gout (*n* = 93)	*P*
Female	11 (35.5)	2 (2.2)	< 0.001*
Age, years	25 (19, 41)	34 (18, 43)	0.001*
Gout symptom onset age, years	18 (10, 29)	28 (14, 41)	0.001*
Family history of HUA	2 (6.5)	16 (17.2)	0.141
Gout symptom duration, years	5.0 ± 4.4	5.0 ± 4.5	0.997
Comorbidities			
Hypertension	0 (0)	28 (30.1)	0.001*
Diabetes mellitus	0 (0)	6 (6.5)	0.335
Hyperlipidaemia	31 (100)	50 (53.8)	< 0.001*
Site of first gouty attack			
Ankle	14 (45.2)	34 (36.6)	0.394
MTP1	12 (38.7)	55 (59.1)	0.048*
Knee	2 (6.5)	3 (3.2)	0.429
Hand joints	2 (6.5)	1 (1.1)	0.092
Elbows	1 (3.2)	0 (0)	0.250
Number of involved joints	3.5 ± 2.0	3.7 ± 2.9	0.053
Upper extremity involved	15 (48.4)	29 (31.2)	0.083
Presence of tophi	15 (48.4)	24 (25.8)	0.019*
Presence of renal calculi	15 (48.4)	20 (21.5)	0.004*
Proteinuria	13 (41.9)	2 (2.2)	< 0.001*
Haematuria	8 (25.8)	1 (1.1)	< 0.001*
Renal insufficiency	2 (6.5)	1 (1.1)	0.092
Maximum value of sUA, umol/L	800.5 ± 131.0	584.3 ± 112.8	0.001*
TG, mmol/L	12.4 ± 10.5	2.9 ± 1.7	0.003*
TC, mmol/L	7.1 ± 2.9	3.6 ± 1.5	0.001*
LDL-C, mmol/L	3.3 ± 1.2	2.8 ± 1.0	0.546
FBS, mmol/L	2.9 ± 0.7	5.5 ± 4.2	0.500
BMI, kg/m^2^	21.4 ± 4.1	27.4 ± 3.4	0.001*

*Abbreviation*: *GSD Ia* glycogen storage disease type Ia, *HUA* hyperuricaemia, *MTP1* first metatarsophalangeal, *SUA* serum uric acid, *TG* triglycerides, *TC* total cholesterol, *LDL-C* low-density lipoprotein cholesterol, *FBS* fasting blood sugar, *BMI* body mass index

^*^P < 0.05

## Discussion

Young patients with HUA should be alert to potential diseases. In addition to well-known secondary causes of HUA, such as myeloproliferative diseases, decreased renal function, and the use of certain drugs, inherited disorders may lead to hyperuricaemia. These include hypoxanthine–guanine phosphoribosyltransferase deficiency, phosphoribosylpyrophosphate synthetase overactivity, familial juvenile hyperuricaemic nephropathy, and various forms of GSD, particularly GSD type I [10]. GSD type Ia is characterized by an abnormal glycogen deposition in the body due to a deficiency in G6Pase activity. Clinical manifestations are usually present at birth or in early infancy but often go undetected without a specific diagnostic evaluation. A protuberant abdomen, hypoglycaemic symptoms, epistaxis, and diarrhoea are the most common symptoms, and HUA, hyperlipidaemia, and hyperlactaemia are almost always present. Early diagnosis and treatment are important for reducing the damaging effects on organs and improving quality of life [22]. Non-invasive molecular genetic testing through gene sequencing of the *G6PC* genes can be used to confirm the diagnosis. *G6PC* contains 5 exons and spans 12.5 kb of DNA on chromosome 17q21. Previous studies involving Chinese patients with GSD Ia showed that c.648G>T and p.R83H were the most prevalent mutations in 112 alleles, whereas p.R83C and p.Q347X were the most prevalent mutations identified in 676 alleles in Caucasian patients [23]. Concerning GSD Ia-related HUA and gout, further research is needed to investigate the differences in genotypes and phenotypes between Chinese patients and patients from other ethnic groups, as well as the relationship between the phenotype and genotype of gout in GSD Ia.

In our study, all adult patients with GSD Ia had HUA, whereas 32.6% had complications of gout. It can be seen that although GSD Ia-related gout is rarely reported in the literature, gout is not uncommon in GSD Ia patients in clinical practice. Additionally, some patients have arthritis as the primary reason for their first-time presentation [24]. If not carefully screened, it is easy to be misdiagnose as primary gout [25], which may affect the growth and development of patients, cause severe complications, and even endanger their lives. Of the 31 patients with GSD Ia-related gout in our cohort, 32.3% of patients were diagnosed with GSD Ia at an average of 5 years after gout onset, further suggesting that awareness of GSD Ia in gout patients needs to be improved. In the present study, the median age of gout onset in GSD Ia patients was 18 years old, which was significantly earlier than that of primary gout, and the youngest age of gout onset was only 10 years old. Meanwhile, the proportion of females was 36.5%, which was significantly higher than that of primary gout. These results indicate that GSD Ia should be screened in early-onset gout patients, especially female patients, and further investigations of clinical manifestations, such as protuberant abdomen, hypoglycaemic symptoms, and growth retardation, may provide relevant clinical clues for the early detection of GSD.

In this study, we found that patients with GSD Ia-related gout had more frequent involvement of upper extremity joints and a higher incidence and earlier onset age of gouty tophi and kidney stones than patients with primary gout. Gouty tophi were identified at 2 years after the first occurrence of articular symptoms. Meanwhile, the sites of gouty tophi were widely distributed among the patients, not only around the joints and auricle but also in the limbs, back, hips, hands, and feet; some patients even needed to undergo surgical removal of the tophi at a young age. Table 5 lists the previously reported cases of GSD Ia-related gout in the English literature in the last 30 years, which shows that 4 out of 11 cases were complicated with gouty tophi [14, 15, 26–31]. In our previous study, we summarized 13 cases of GSD Ia-related gout reported in China and found that 9 cases were complicated with gouty tophi [11]. Among them, two patients underwent surgical removal of the tophi, one due to giant tophus in the hips (17 cm × 12 cm × 6 cm in the left and 10 cm × 8 cm × 4 cm in the right) and the other due to a giant tophus on the feet with an ulcer that does not heal. It can be observed that GSD Ia-associated gout patients have earlier, more frequent, and widely distributed gouty tophi, which may lead to serious complications and even require surgical treatment if not treated in a timely manner. The long course of HUA in GSD Ia patients may explain the earlier occurrence and the abnormally extensive deposition of gouty tophi. Considering that several studies have reported that some patients with asymptomatic HUA have subclinical MSU crystal deposits [32, 33], early joint ultrasound screenings may be helpful in the early detection of urate deposition or early joint lesions in GSD Ia patients, although this requires further study.

**Table 5:** Reported cases of gout in glycogen storage disease type Ia in the recent 30 years

Reference	Sex	Age	Age of gout onset	Sites of gouty attacks	Gouty tophi	Renal involvement	Other presentations	SUA (μmol/L)	LA (mmol/L)	TC (mmol/L)	TG (mmol/L)	FBS (mmol/L)	ULT
Talente et al. [14]	M	43	18	–	–	Renal medullary calcifications. Renal parenchymal cysts. Renal biopsy showed focal glomeru losclerosis. Progressive renal insufficiency requiring chronic haemodialysis.	Hepatomegaly, multiple hepatic adenomas, growth retardation, frequent nosebleeds	339	–	8.22	17.5	2.8	Allopurinol
Hou et al. [26]	F	14	14	–	Subcutaneous nodules over the dorsum of right hand	–	Hepatomegaly, growth retarcation, abnormal liver function	916	5	5.04	8.77	2.2	–
Faivre et al. [27]	M	23	21	MTP1	–	–	Hepatomegaly, multiple hepatic adenomas, growth retardation	711	↑	–	–	↓	Allopurinol
Carvès et al. [24]	M	17	17	Right Achilles tendon	–	–	Hepatomegaly	680	7	–	3.26	3.5	Allopurinol
Carvès et al. [24]	F	14	14	–	–	Proteinuria (24-h urinary protein was 1.475 g).	Hepatomegaly	625	4.42	6.14	3.3	–	–
Zhang et al. [28]	F	23	20	Left MTP1, bilateral ankle, and knee joints	–	Renal biopsy showed chronic interstitial tubular change.	Hepatomegaly, growth retardation, abnormal liver function, osteoporosis, mild anaemia	567	–	9.35	13.08	3.1	Anti-hyperuricaemic drugs
Adenwalla et al. [29]	F	37	30	Catastrophic axial gout that caused paraplegia	Extensive tophaceous deposits involving her bilateral pinnae, elbows, and interphalangeal joints of upper and lower extremities	–	Hepatomegaly, abnormal liver function, mild anaemia	648	–	–	2.37	–	Febuxostat
Ng et al. [30]	M	28	16	Large and small joints of all 4 limbs	Extensive tophaceous deposits involving MCP, interphalangeal joints, forearms, elbows, and the pinna of his ears	–	Hepatomegaly, hepatic adenoma, severe anaemia	830	10.2	–	2.14	2.9	*–*
Shieh et al. [31]	M	32	21	–	–	–	Hepatomegaly, hepatic hemangioma, mild anaemia	690	4.12	2.10	3.52	3.6	Allopurinol
Shieh et al. [31]	F	34	18	–	–	–	Hepatomegaly, mild anaemia	534	2.49	3.04	10.45	3.7	Allopurinol
Zhang et al. [15]	F	27	13	MTP joints, bilateral ankles, right knee	Multiple nodules in her MTP joints, ankles, and fingers	Renal medullary calcifications.	Hepatomegaly	789	7.4	6.6	6.22	3.6	Allopurinol and benzbromarone

*Abbreviation*: *MCP* metacarpophalangeal, *SUA* serum uric acid, *LA* lactic acid, *TG* triglycerides, *TC* total cholesterol, *LDL-C* low-density lipoprotein cholesterol, *FBS* fasting blood sugar, *ULT* urate-lowering therapy

“–”, no records

In addition, chronic renal disease is a common complication of GSD Ia, especially in adult patients [34]. Kidney enlargement due to glycogen deposition is common [35]. As a result of the metabolic perturbations, glycogen accumulation, and prolonged glomerular hyperfiltration in cases with insufficient metabolic control, progressive glomerular injury with proteinuria and/or haematuria can develop in addition to proximal and distal renal tubular dysfunction [36, 37]. This can further result in renal dysfunction and even end-stage renal disease requiring renal replacement therapy [37, 38]. It should be noted that persistently elevated SUA levels and renal stones in themselves have both been implicated in causing or accelerating renal injury [39, 40]. Optimal treatment for HUA instituted early in life may also be helpful to prevent, delay, or slow the progression of renal disease.

In the present study, the mean level of maximum SUA in GSD Ia-related gout patients was 800.5 μmol/L, which is notably higher than that in primary gout patients. The incidence of gout in adult GSD Ia patients significantly correlates with the initial age of regular treatment with raw corn starch, the proportion of ULT initiated during the asymptomatic HUA stage, SUA level, and cholesterol level; thus, the importance of early comprehensive management of metabolic disorders is emphasized. Moreover, the early identification and diagnosis of GSD Ia and the subsequent early initiation of treatment with raw corn starch in infancy may help prevent or partially improve the high levels of uric acid that can cause gout. There is no consensus on whether or when ULT should be initiated for asymptomatic hyperuricaemia in GSD Ia patients. For primary asymptomatic hyperuricaemia, a few guidance documents recommended a “no pharmacological” treatment under any circumstances, whereas some documents recommended ULT treatments in asymptomatic hyperuricaemic patients with comorbidities, or with extremely high SUA levels [41, 42]. The 2020 ACR guidelines conditionally recommend against initiating ULT in patients with asymptomatic hyperuricaemia [43]. However, for patients with GSD Ia-related asymptomatic hyperuricaemia, relatively high SUA levels were mostly presented since infancy or childhood, and our results showed that almost one-third of adult patients were complicated with early-onset gout, a marker of gout severity that should prompt earlier treatment [44, 45]. This finding suggests that early initiation of ULT in addition to raw corn starch, a low-purine diet, and lifestyle interventions in the period of asymptomatic HUA may help reduce the risk of gout, prevent severe complications, and improve prognosis. Further investigation into the timing of initiating ULT for asymptomatic hyperuricaemia in GSD Ia patients is needed.

Recurrent hypoglycaemia causes lactic acidosis, hepatomegaly, hypertriglyceridaemia, and hyperuricaemia; good glucose control improves several of the metabolic sequelae of GSD Ia. Raw corn starch has been used as the first-line treatment in GSD-Ia patients [46] and may maintain normoglycaemia for up to 6 h. Small, frequent meals comprising complex carbohydrates, in addition to cornstarch therapy, is important for preventing hypoglycaemia. Diet adherence and good metabolic control from the onset may prevent or reduce high levels of uric acid that can cause gout [47]. Because of the possibility of asymptomatic hypoglycaemia, regular blood glucose monitoring is essential for a well-controlled GSD. The aim should be to keep blood glucose at ≥ 70mg/dL and to avoid rapid glucose fluxes [47–49]. However, with intensive dietary treatment, clinical and biochemical statuses improve but cannot be completely corrected in most patients, as more complications develop with ageing. Thus, metabolic disorders, such as HUA and hyperlipidaemia, need to be controlled during the disease course. Liver transplantation may be considered in patients who have not had success with medical management, have developed hepatic adenomas, and are at a high risk for liver cancer [47, 50]. However, due to the risk of complications associated with transplantation, it is not recommended that GSD Ia patients receive routine liver transplantation. Gene therapy is still in the exploratory stage, which may be a promising treatment for GSD I in the future [51].

For patients with GSD Ia-related gout, there is no consensus on when to start ULT and what serum urate target to achieve. According to the 2020 ACR guidelines for primary gout [43], pharmacologic ULT should be considered for gout patients with subcutaneous tophi, evidence of radiographic damage attributable to gout, or frequent gout flares (≥ 2 /year). Achieving and maintaining an SUA target of < 6 mg/dL (360 μmol/L) is recommended for all patients receiving ULT. However, high levels of SUA and metabolic interaction in patients with GSD Ia-related gout may increase the difficulty of treat-to-target with ULT in clinical practice; the serum urate target in GSD Ia-related patients remains to be further studied. Allopurinol is recommended as a first-line ULT for gout in GSD Ia patients, and it is the most commonly used therapy in previously reported cases. Febuxostat can be considered in patients who are unable to use allopurinol, those with extremely high serum urate levels, and in the presence of refractory tophi [52]. It is very difficult to treat patients with chronic gout that is refractory to conventional therapy, and the therapeutic options are limited. Pegloticase, a pegylated recombinant uricase, is a novel agent that is approved in the US for adult patients with chronic refractory gout. Some studies have demonstrated the efficacy of pegloticase in markedly lowering serum urate levels, resolving tophi, and improving functional status and quality of life [53–55]. Pegloticase is recommended for patients with gout in whom xanthine oxidase inhibitor treatment, uricosurics, and other interventions have failed to achieve the SU target and who have recurrent gout flares or non-resolving subcutaneous tophi [43]. Owing to pegloticase immunogenicity and the emergence of anti-drug antibodies, which are associated with the risk of infusion reaction and loss of drug efficacy, in making a decision regarding pegloticase use, the potential clinical benefits and problems (including costs) should be carefully weighed. Moreover, there are no studies on the efficacy and safety of pegloticase for the treatment of chronic refractory gout in GSD Ia. Due to the lack of literature reports, the SUA target of ULT, efficacy, and prognosis still needs to be further elucidated. Meanwhile, in the present study, the adherence to ULT in GSD Ia-related gout patients was poor, indicating that continuous education of lifestyle changes and ULT should be strengthened among GSD Ia-related gout patients.

There were some limitations in this study. First, our findings were based on the results of a single-centre, retrospective study. Second, nationality and race were limited to Chinese individuals. Therefore, there is limited generalizability, and the possibility of selection bias cannot be excluded. Third, because GSD is a rare disease, the number of the patients involved in this study was relatively small, and clinical features of gout and non-gout in GSD Ia were compared based on a relatively small number of subjects. A large-scale prospective study is required to further explore the risk factors and long-term prognosis of gout in GSD Ia patients.

## Conclusion

The incidence of gout in adult patients with GSD Ia is common. Determination of the presence of primary disease should be performed for patients with young-onset gout with an early occurrence of gouty tophi. GSD type I should be suspected if there are existing clinical manifestations such as hepatomegaly, recurrent hypoglycaemia, or growth retardation. Close SUA monitoring from the early stage of disease and early intervention of HUA should be emphasized in GSD Ia patients. Early urate-lowering treatment as clinically indicated, in addition to raw corn starch and lifestyle intervention, should be implemented to maintain good metabolic control. This may help to reduce the risk of gout, or prevent, or delay the development of subsequent complications.

## Data Availability

All data generated or analysed during this study are included in this published article.

## Abbreviations

eGFR: Estimated glomerular filtration rate
EULAR: European League Against Rheumatism
G6Pase: Glucose-6-phosphatase
GSD Ia: Glycogen storage disease type Ia
HUA: Hyperuricaemia
MCP: Metacarpophalangeal
MTP1: First metatarsophalangeal
PIP: Proximal interphalangeal
SD: Standard deviation
SUA: Serum uric acid
ULT: Urate-lowering therapy
